# Landscape genetic structure and evolutionary genetics of insecticide resistance gene mutations in *Anopheles sinensis*

**DOI:** 10.1186/s13071-016-1513-6

**Published:** 2016-04-23

**Authors:** Xuelian Chang, Daibin Zhong, Eugenia Lo, Qiang Fang, Mariangela Bonizzoni, Xiaoming Wang, Ming-Chieh Lee, Guofa Zhou, Guoding Zhu, Qian Qin, Xiaoguang Chen, Liwang Cui, Guiyun Yan

**Affiliations:** Anhui Key Laboratory of Infection and Immunity, Bengbu Medical College, Bengbu, Anhui 233000 China; Program in Public Health, College of Health Sciences, University of California at Irvine, Irvine, CA 92697 USA; Key Laboratory of Prevention and Control for Emerging Infectious Diseases of Guangdong Higher Education Institutes, School of Public Health and Tropical Medicine, Southern Medical University, Guangzhou, Guangdong 510515 China; Department of Entomology, Pennsylvania State University, University Park, PA 16802 USA

**Keywords:** Para-type sodium channel gene, Mitochondrial DNA, Evolution, Mutation, Knockdown resistance, *Anopheles sinensis*

## Abstract

**Background:**

*Anopheles sinensis* is one of the most abundant vectors of malaria and other diseases in Asia. Vector control through the use of insecticides is the front line control method of vector-borne diseases. Pyrethroids are the most commonly used insecticides due to their low toxicity to vertebrates and low repellency. However, the extensive use of insecticides has imposed strong selection pressure on mosquito populations for resistance. High levels of resistance to pyrethroid insecticides and various mutations and haplotypes in the *para* sodium channel gene that confers knockdown resistance (*kdr*) have been detected in *An. sinensis*. Despite the importance of *kdr* mutations in pyrethroid resistance, the evolutionary origin of the *kdr* mutations is unknown. This study aims to examine the evolutionary genetics of *kdr* mutations in relation to spatial population genetic structure of *An. sinensis*.

**Methods:**

Adults or larvae of *Anopheles sinensis* were collected from various geographic locations in China. DNA was extracted from individual mosquitoes. PCR amplification and DNA sequencing of the *para-type* sodium channel gene were conducted to analyse *kdr* allele frequency distribution, *kdr* codon upstream and downstream intron polymorphism, population genetic diversity and *kdr* codon evolution. The mitochondrial cytochrome *c* oxidase COI and COII genes were amplified and sequenced to examine population variations, genetic differentiation, spatial population structure, population expansion and gene flow patterns.

**Results:**

Three non-synonymous mutations (L1014F, L1014C, and L1014S) were detected at the *kdr* codon L1014 of *para-type* sodium channel gene. A patchy distribution of *kdr* mutation allele frequencies from southern to central China was found. Near fixation of *kdr* mutation was detected in populations from central China, but no *kdr* mutations were found in populations from southwestern China. More than eight independent mutation events were detected in the three *kdr* alleles, and at least one of them evolved multiple times subsequent to their first divergence. Based on sequence analysis of the mitochondrial COI and COII genes, significant and large genetic differentiation was detected between populations from southwestern China and central China. The patchy distribution of *kdr* mutation frequencies is likely a consequence of geographic isolation in the mosquito populations and the long-term insecticide selection.

**Conclusion:**

Our results indicate multiple origins of the *kdr* insecticide-resistant alleles in *An. sinensis* from southern and central China. Local selection related to intense and prolonged use of insecticide for agricultural purposes, as well as frequent migrations among populations are likely the explanations for the patchy distribution of *kdr* mutations in China. On the contrary, the lack of *kdr* mutations in Yunnan and Sichuan is likely a consequence of genetic isolation and absence of strong selection pressure. The present study compares the genetic patterns revealed by a functional gene with a neutral marker and demonstrates the combined impact of demographic and selection factors on population structure.

**Electronic supplementary material:**

The online version of this article (doi:10.1186/s13071-016-1513-6) contains supplementary material, which is available to authorized users.

## Background

The development of insecticide resistance is regarded as an empirical model of an adaptive trait [1] whose origin and spread depend upon the selection pressure applied to the target population, the fitness cost associated with the resistant allele and the gene flow among populations [2]. Geographical landscape is often considered as an important factor limiting gene flow among populations [3–6]. However, the impact of gene flow barriers imposed by landscape differences and local selection on the spatial distribution of resistance alleles is largely unknown.

Resistance to insecticides in mosquitoes involves multiple mechanisms, including the target-site insensitivity caused by mutations in the *para* sodium channel gene (knockdown resistance *kdr*) and the detoxification of insecticides in mosquitoes involving detoxification enzymes (i.e. P450 monooxygenases, glutathione-S-transferases and esterases) that metabolise the insecticide before it reaches its target (metabolic resistance) [7–19]. Mutations in the *para* sodium channel gene have been shown to be associated with the use of pyrethroids, the most commonly used class of insecticide for malaria control worldwide [13]. The spectrum of mutations at the *para* sodium channel gene is highly conserved across insect species, indicating convergent evolution [11]. There are two hypotheses that relate to the distribution of *kdr* mutations: either the mutations arose once and then spread, or have evolved independently in different populations across a broad landscape. For instance, in *Anopheles gambiae*, the main African malaria vector, non-synonymous mutations at position L1014 of the *para* sodium channel gene occurred at least 4–5 times independently [1, 20]. Some of the mutation events at the *para* sodium channel gene were derived from single mutational steps from the common ancestor whereas others were the result of two mutational steps [1, 20]. Multiple origins of *kdr* mutations were also found in *Musca domestica* and in the aphid *Myzus persicae* [21, 22]. An alternative situation was seen in the agricultural pest *Bemisia tabaci*, where the L925I and T929V mutations appeared to have originated once and then spread through migration and global trade [2, 23].

In *An. sinensis*, the most abundant and important malaria vector species in southeast Asia [24–32], four non-synonymous mutations at codon L1014 of the *para* sodium channel gene are known, including the L1014F [31, 33–37], L1014S [17, 35], L1014C [31, 33, 34, 36, 37] and L1014W [35] mutations. There could be various factors influencing the distribution of *kdr* allele variants in *An. sinensis* across China and Southeast Asia. For example, the complexity of landscape could impose barriers to gene flow between the mosquito populations. Selection based upon the intensity and duration of pyrethroid use could also determine allelic distribution of *kdr.* On the other hand, genetic variation based on neutral markers reflects population structure resulting from demographic factors. In this case, the cytochrome *c* oxidase genes of the mitochondrial genome are used as the neutral reference. Based on the unique characteristics of maternal inheritance, no recombination, high variability relative to nuclear DNA, and low effective population size, the mitochondrial DNA (mtDNA) has been a marker of choice for studies of genetic diversity and population structure.

The central goal of this study was to understand how local selection and migration have shaped the distribution of *kdr* mutations. The specific objectives were to (i) determine the *kdr* allele distribution in multiple sites over a large geographic region; (ii) elucidate the evolutionary origin(s) of *kdr* mutations; and (iii) examine the role of landscape and demographic history of *An. sinensis* on the evolution of *kdr* mutations using mitochondrial DNA sequence data.

## Methods

### Sample collection and preparation

**Fig. 1:**
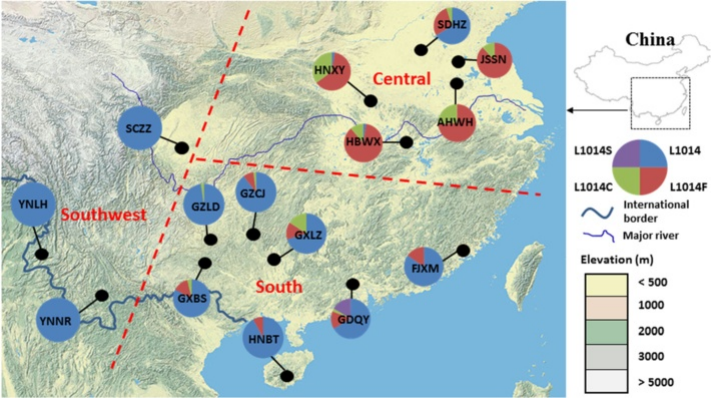
Pie charts showing *kdr* allele frequency distribution of *Anopheles sinensis* in China. Site names and abbreviations are as in Table 1

*Anopheles sinensis* mosquitoes were collected from 15 localities in China, including five sites in central China, seven sites in south China and three sites in southwest China (Fig. 1). For each site, approximately 50 female adult mosquitoes were collected with three to five CDC light-traps. For two sites that had an insufficient number of adult mosquitoes, approximately 50 larval mosquitoes were collected from 20 breeding sites (irrigated rice fields and small ponds with aquatic plants) using the standard 350 ml dippers. The larvae were then reared to adults and identified as *An. sinensis* based on the published morphological keys [38]. Between 13 and 36 mosquitoes per population were used for DNA analysis, giving a total of 344 mosquitoes (Table 1). Genomic DNA was extracted from each sample following a standard phenol:chloroform method [39]. DNA was re-suspended in TE buffer (10 mM Tris, 0.1 mM EDTA, pH 7.4). Mosquito species identity was further confirmed by amplifications of the ribosomal internal transcribed spacer ITS2 of the nuclear rRNA gene using specific primers for *An. sinensis* [40, 41].

**Table 1:** Summary of *Anopheles sinensis* specimen collection sites in China

Site ID	Name abbreviation	Province: Locality	Latitude (N)	Longitude (E)	Elevation (m)	Collection date	Life-stages analysed	Specimen genotyped
1	YNLH	Yunnan: Lianghe	24°51’	98°18’	1199	12-Jun	adult	26
2	YNNE	Yunnan: Ninger	23°03’	101°02’	1426	7-Jul	adult	24
3	SCNJ	Sichuan: Neijiang	29°40’	104°57’	321	11-Aug	adult & larva	22
4	GXBS	Guangxi: Baise	23°23’	105°49’	1072	12-Aug	adult	27
5	GZLD	Guizhou: Luodian	25°25’	106°45’	830	12-Aug	adult	21
6	GZCJ	Guizhou: Congjiang	25°44’	108°54’	566	12-Aug	adult	27
7	GXLZ	Guangxi: Liuzhou	25°45’	109°36’	352	12-Aug	adult	21
8	HNBT	Hainan: Baoting	18°38’	109°46’	120	12-Jun	adult	12
9	GDQY	Guangdong: Qingyuan	23°40’	113°03’	87	12-Jun	adult	20
10	FJXM	Fujian: Xiamen	24°25’	118°24’	66	12-Aug	adult & larva	22
11	HBWX	Hubei: Wuxue	29°50’	115°33’	14	11-Jul	adult	23
12	HNXY	Henan: Xinyang	32°06’	114°01’	117	11-Jul	adult	22
13	JSSN	Jiangsu: Suining	33°52’	117°59’	24	11-Jul	adult	32
14	AHWH	Anhui: Wuhe	33°09’	117°51’	17	12-Jul	adult	22
15	SDHZ	Shandong: Heze	34°46’	116°06’	44	12-Aug	adult	23

### Amplification and sequencing of the mitochondrial cytochrome *c* oxidase genes

The COI and COII genes of the mitochondrial genome were amplified, respectively, using degenerate primers 1809 F (5'-CMC TTT CAT CTG GAA TTG CT-3') and 2708R (5'-AAA AAT GTT GAG GGA ARA ATG TTA-3') for COI (~900 bp) and primers COIIF (5'-TCT AAT ATG GCA GAT TAG TGC A-3') and COIIR (5'-ACT TGC TTT CAG TCA TCT AAT G-3') for COII (~800 bp). Reaction mix consisted of 10.5 μl 2X SYBR® Green PCR Master Mix (Thermo Fisher Scientific Inc., Waltham, MA, USA), 1 μl of template DNA (5–20 ng), 0.5 μl of 10 μM each primer, and 8.5 μl water. Conditions for polymerase chain reaction (PCR) were 95 °C for 3 min, 35 cycles of 94 °C for 30 s, 55 °C for 30 s, and 72 °C for 60 s, followed by an additional extension step at 72 °C for 6 min. Products of PCR were visualised on 1 % agarose gel and then purified and sequenced from both ends with the ABI Big Dye Terminator Cycle Sequencing Kit.

### Amplification and sequencing of a 1285 bp fragment of the *para* sodium channel gene

We designed a pair of primers (Forward primer 63 F: 5'-GAC GTT CGT GCT CTG CAT TA-3', Reverse primer 1347R: 5'-GAG CGA TGA TGA TCC GAA AT-3') based on the *An. sinensis* sodium channel gene sequence (GenBank accession: DQ334052) to amplify a 1285 bp fragment that encompasses the *kdr* target codon position L1014, an upstream intron (intron-1) and a downstream intron (intron-2). Both primers are located in the coding region. Two internal primers, 566 F (5'-CAC TGC TGT AAA ACC CTG TGT-3') and 855R (5'-CTG TTT GCT AGG CAG TTT GC-3') were used for sequencing. We amplified the 1285 bp fragment from individual specimen and a total of 273 specimens were examined. Reaction mix consisted of 10.5 μl 2X SYBR® Green PCR Master Mix (Thermo Fisher Scientific Inc., Waltham, MA, USA), 1 μl of template DNA (5–20 ng), 0.5 μl of 10 μM each primer, and 8.5 μl water. PCR cycles involved an initial denaturing step at 95 °C for 3 min, 35 cycles of 94 °C for 1 min, 55 °C for 1 min, and 72 °C for 2 min, followed by an additional extension step at 72 °C for 6 min. Amplifications were performed in a Bio-Rad MyCycler Thermal Cycler. Products of PCR were visualised on 1 % agarose gel and then purified and sequenced from both ends with ABI Big Dye Terminator Cycle Sequencing Kit.

### Data analysis

This study addressed the central question as to whether the patchy *kdr* allele distribution in *An. sinensis* populations from southern China to central China was shaped by geographic isolation and/or by insecticide selection. We asked two questions in particular, i.e., (i) What is the *kdr* allele frequency distribution pattern and the phylogenetic relationship of *kdr* haplotypes? and (ii) What is the spatial genetic structure of *An. sinensis* mosquito populations in China? We used the *para* sodium channel gene sequence data to address the first question used mtDNA sequence data to address the second.

#### Phylogenetic relationships of *kdr* haplotypes

Sequenced 1285 bp fragments of the *para* sodium channel gene encompassing the *kdr* codon L1014, intron-1 and intron-2 were aligned in Bioedit version 7.0 [42]. DNA polymorphism including the number of segregating sites, number of haplotypes, haplotype diversity and nucleotide diversity were calculated using DnaSP v5 [43]. The two intron regions were tested for neutrality by Tajima‘s *D* [44], Fu and Li’s D* and F* [45] and F tests [46]. Partition of the genetic variation among and within populations was examined by AMOVA test implemented in Arlequin v3.5 [47]. A Bayesian approach based on a priori predictions from the coalescent theory implemented in Phase 2.1. [48] was used to reconstruct haplotypes from the population genotypic data in mosquitoes heterozygous at more than one site within the amplified 1285 bp fragment. These haplotypes were confirmed by cloning of the 1285 bp fragment in a subset of 20 individuals. Amplified PCR products from the selected 20 individuals were cloned into pCR2.1TOPO TA vectors (Invitrogen, Carlsbad, CA, USA). Three to five clones were sequenced for each individual to identify haplotypes.

A statistical parsimony network was constructed using the TCS v1.21 software [49] to infer the genealogical relationships among haplotypes based on an analysis of 1285 bp fragments in the samples. The software calculates the frequencies of the haplotypes in the sample and estimates haplotype outgroup probabilities, which correlate with haplotype age [50]. The pairwise comparisons of haplotypes was conducted using the parsimony algorithm described in Templeton et al. [51] to estimate the number of mutational steps between haplotypes using a connection limit at two steps and a connection probability threshold of 0.95.

#### Spatial genetic structure of *An. sinensis*

COI and COII gene sequences were aligned using Bioedit version 7.0 [42]. The number of variable sites and haplotypes, as well as haplotype and nucleotide diversity were estimated using Arlequin v3.5 [47] and DnaSP v5 [43]. To determine the genetic structure of the mosquito populations, we used analysis of molecular variance (AMOVA) to partition genetic variation among groups (*F*_CT_), among populations within groups (*F*_SC_) and among populations among groups (*F*_ST_). Pairwise *F*_ST_ and *Ф*_ST_ among all populations were calculated. Significance values were adjusted for multiple comparisons using the false discovery rate (FDR) approach [52]. Pairwise genetics distances were used to construct unrooted UPGMA tree in Phylip 3.695 [53] to infer population clustering. Isolation-by-distance among populations was explored with Mantel tests [54] using the Isolation by Distance Web Service v3.23 [55]. Euclidian geographical distances among the 15 populations were calculated based on geographical coordinates using great circle calculations to accurately represent distances on a spherical earth. Pairwise genetic distance between populations was calculated as *F*_ST_/(1- *F*_ST_) with 10,000 randomizations [56].

To examine the proportion of total genetic variance between groups of populations and identify possible genetic barriers between them, the spatial genetic structure of haplotypes (combined COI and COII sequences) was analysed using the Spatial Analysis of Molecular Variance (SAMOVA 2.0 software) [57]. This software implements an approach, which combines genetic differentiation and geographical distance to define groups of populations that are maximally differentiated from each other, without the constraint for the geographic composition of the groups. The number of initial conditions was set to 100 simulated annealing processes for each configuration of K groups, with K ranging from 2 to 10, and run for 10,000 iterations. For each K, the configuration with the largest *F*_CT_ values after the 100 independent simulated annealing processes was retained as the best grouping of populations.

#### Genetic landscape shape (GLS) interpolation analysis

We performed the genetic landscape shape (GLS) interpolation analysis based on the pairwise genetic and geographical distance matrices using the Alleles-In-Space (AIS) software [58]. AIS is a computer program for the joint analysis of inter-individual spatial and genetic information. Detailed patterns of spatial genetic structure across the complete sampled area were visualised using the ‘genetic landscape shape’ interpolation procedure. This procedure produced a 3-dimensional surface plot where X- and Y-axes correspond to geographical locations and surface heights (Z-axes) represent genetic distances. Genetic structure across the landscape was inferred from measured genetic distances using an inverse distance weighted interpolation across a uniform grid laid over the entire sampling area. GLS was made by using a 80 × 80 grid and a raw genetic distance, with a distance weighting parameter α = 1.

#### Demographic history

Demographic history of the 15 populations was elucidated based on the neutrality indices Tajima’s *D*, Fu’s *F*s and Ramos-Onsins and Rozas’s R2 statistic in DnaSP. Significantly negative *D*, *F*s or small R2 values indicated recent population expansion, whereas positive or high values indicated population decline. Population expansion parameter τ (τ = 2 t* μ; where t = time in years, μ = mutation rate per locus) was estimated using the moment method, assuming the infinite sites model [59]. Deviations from a model of population expansion were evaluated by computing the statistical significance of sums of squared deviation (SSD) and Harpending's raggedness index (*r*) over 1000 simulated samples of pairwise nucleotide differences. All *P*-values were corrected for multiple testing using the false discovery rate (FDR) approach [52].

#### Migration rate between populations

Gene flow and migration rates between all pairwise populations were estimated using the Bayesian coalescence-based approach implemented in LAMARC version 2.1.10 [60]. Felsenstein‘84 (F84) substitution model was used with empirical base frequencies [61, 62]. Default prior settings were used for Theta and Bayesian estimation of migration rates. LAMARC analysis consisted of 3 simultaneous searches with automatically adjusted heating temperature using 10 initial chains, with 500 samples and a sampling interval of 20 steps, followed by 2 final chains, with 10,000 samples and sampling interval of 20 steps. One thousand samples were discarded for initial and final burn-in. Migration rate was measured as 4 Nm, a multiplication of LAMARC‘s M and Theta values for the recipient population.

## Results

The PCR amplification of *para-type* sodium channel gene generated a 1285 bp PCR fragment, including a 187 bp exon encompassing the *kdr* target codon position L1014, its upstream intron 905 bp and exon 74 bp, and its downstream intron 64 bp and exon 55 bp. The PCR amplification of the COI and COII genes of the mitochondrial genome resulted in 900 bp and 774 bp fragments, respectively. In order to elucidate the *kdr* allele frequency distribution pattern and the phylogenetic relationships of *kdr* haplotypes, the para sodium channel gene sequence data were analysed for *kdr* allele frequency distribution, *kdr* codon upstream and downstream intron polymorphism, population genetic diversity and *kdr* codon evolution. Because mtDNA is haploid and maternally inherited, isolated populations will drift to different haplotype frequencies faster and achieve approximately twice the level of differentiation comparing to nuclear markers. So, the mtDNA sequence data were analysed for population variations, genetic differentiation, spatial population structure, population expansion and gene flow patterns.

### *Kdr* allele frequency distribution

Three non-synonymous mutations (L1014F, L1014C, and L1014S) were detected at the *kdr* codon L1014. Overall, the distribution of *kdr* mutant allele frequencies varied by geographic location. Populations in central China (Anhui, Hubei, Hunan and Jiangsu) reached a near fixation for *kdr* at codon L1014 mutations, whereas the southern populations (Guangdong, Fujian, Guangxi, Hainan and Guizhou) exhibited a low frequency of *kdr* mutations (Fig. 1). We did not detect any *kdr* mutation in the southwestern populations (Yunnan and Sichuan provinces). In central China, L1014F was the predominant mutation and L1014C was also common in three provinces (Henan, Hubei and Anhui) in central China (Fig. 1).

### *Kdr* codon upstream and downstream intron polymorphism

The 1285 bp fragment examined encompasses the *kdr* target codon position L1014, an upstream intron (intron-1), and a downstream intron (intron-2). Among the 344 total samples, 273 samples were sequenced, and a total of 71 polymorphic sites were observed. Of these sites, 69 were bi-allelic and two (at the second and third nucleotides of the *kdr* codon L1014) had three variants (Table 2). The two polymorphic sites with three variants were located at nucleotide positions 1161 and 1162 in a template sequence (GenBank KP763726). In intron-1, we observed deletions at nucleotide positions 946 and 949, and one-two base insertions at nucleotide position 230 when compared with a reference sequence (GenBank DQ334052). Among the 71 polymorphic sites, six were located in the exon harboring the *kdr* codon L1014, 58 were in intron-1, and seven in intron-2. Because of the heterozygous genotypes at the polymorphic sites, haplotypes were predicted by the Phase analysis [63, 64]. The Phase analysis identified a total of 85 haplotypes in 71 polymorphic sites (GenBank KP763726–KP763810). We cloned *kdr* fragments from 20 individuals with polymorphic sites at the 1285 bp *kdr* fragment and sequenced a total of 100 clones. Cloned sequences confirmed the haplotypes predicted by the Phase analysis. The 65 polymorphic sites in intron-1 and intron-2 yielded a total of 72 haplotypes.

**Table 2:** Polymorphism of *kdr* intron and neutrality test of 15 *Anopheles sinensis* populations

Pop	n	S	K	Pi	Tajima’s *D*	h	*H*d	Fu’s *Fs*	Fu and Li’s D*	Fu and Li’s F*	τ	SSD	r
Southwest													
YNLH	42	16	4.570	0.070	0.731	14	0.913	−1.598	0.337	0.553	5.486	0.032*	0.114*
YNNE	32	18	4.776	0.073	0.233	13	0.917	−1.637	0.909	0.815	5.496	0.025	0.095*
SCNJ	44	15	3.744	0.058	0.269	16	0.922	−4.107*	0.678	0.640	5.066	0.011	0.029
South													
GXBS	22	16	4.368	0.067	−0.018	17	0.974	−9.984**	−0.135	−0.116	4.879	0.004	0.018
GZLD	32	7	3.601	0.055	3.118	4	0.575	5.319*	1.269	2.138*	8.051	0.260	0.541**
GZCJ	40	15	4.318	0.066	0.715	18	0.924	−5.611*	0.698	0.830	5.539	0.004	0.015
GXLZ	32	11	3.216	0.049	0.563	14	0.823	−4.616*	−0.092	0.132	5.156	0.023	0.088
HNBT	30	13	3.724	0.057	0.445	13	0.871	−3.066*	0.174	0.303	5.830	0.030	0.058
GDQY	36	15	5.335	0.082	1.543	11	0.883	0.529	1.137	1.493	6.641	0.021	0.071
FJXM	26	17	4.028	0.062	−0.338	14	0.923	−4.397*	−0.463	−0.497	2.371	0.003	0.010
Central													
HBWX	42	6	1.153	0.018	−0.457	4	0.301	1.111	0.356	0.122	3.000	0.056*	0.497
HNXY	38	5	0.906	0.014	−0.614	4	0.368	0.377	0.204	−0.052	3.000	0.038	0.310
JSSN	30	6	0.759	0.012	−1.424	4	0.251	−0.257	−1.111	−1.403	3.000	0.030	0.511
AHWH	62	6	0.754	0.012	−1.001	5	0.292	−0.700	1.158	0.544	3.000	0.030	0.425
SDHZ	38	29	9.757	0.150	1.431	9	0.728	5.974*	0.770	1.177	18.506	0.080*	0.118*

*n* number of sequences used, *S* number of polymorphic (segregating) sites, *K* average number of pairwise nucleotide differences, *Pi* nucleotide diversity, *h* number of Haplotypes, *Hd* haplotype diversity, *τ* estimated parameter of population expansion assuming the stepwise growth model (*τ* = 2 t*MU where t = time in years, MU = mutation rate per locus), *SSD* the sum of squared deviations; and *r* Harpending’s raggedness index

*P < 0.05; **P < 0.01 (FDR < 0.02)

The overall haplotype diversity (*H*d) and nucleotide diversity (Pi) were 0.82 and 0.07, respectively. Genetic diversity varied significantly among geographical regions (Table 2). In central China, four of the five populations showed low haplotype (0.30 ± 0.05; *F*_(2,11)_ = 46.3, *P* < 0.0001) and nucleotide diversity (0.014 ± 0.003, *F*_(2,11)_ = 47.3, *P* < 0.0001), whereas in the south and southwest populations, high haplotype (0.92 ± 0.005 and 0.85 ± 0.13) and nucleotide diversity (0.07 ± 0.005 and 0.06 ± 0.03) were detected.

The Tajima’s *D* and Fu and Li's D* tests indicated no significant departures from neutrality in all the populations. Similarly, only one test of Fu and Li‘s F* indicated significant departure from neutrality in all the populations. However, significant departures from neutrality were detected by Fu’s *Fs* in eight populations and seven of them were from the south and southwest (Table 2), which indicated a pattern of haplotype diversity that deviated from the expectation under neutrality. Since the Fu‘s *Fs* statistic is particularly sensitive to demographic effects, it is difficult to conclude whether positive selection or demographic history (e.g., population expansion) account for the observed pattern. Although the AMOVA indicated that most of the variation (~70 %) occurred among populations among regions (*F*_ST_ = 0.28, *P* < 0.001), the percentage of variation found among populations within the same region (3.2 %) as well as among the three regions (27.9 %) was also highly significant (*F*_SC_ = 0.04, *P* < 0.001; *F*_CT_ = 0.18, *P* < 0.001). Both the *F*_ST_ and *Ф*_ST_ values (based only on haplotype frequencies) were significant for 61 and 55 out of the 105 pairwise comparisons, respectively, after Bonferroni correction for multiple comparisons (Additional file 1: Table S1). The majority of the differences were detected between the central China populations and the remaining populations.

### *kdr* codon evolution

**Fig. 2:**
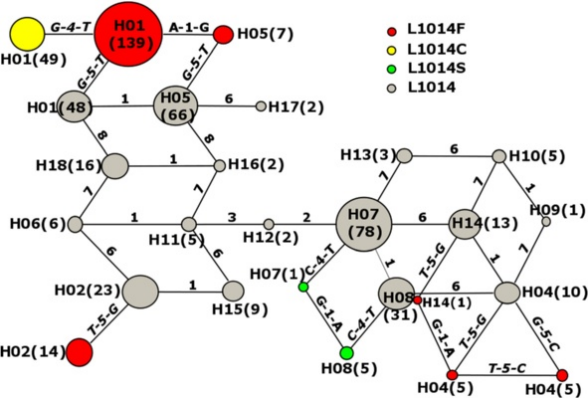
*kdr* haplotype networks showing the genealogical relationship for *Anopheles sinensis*. Each haplotype is represented by a circle with size proportional to its frequency in the sample (sample size in parentheses, 2 N = 546). Mutational steps are represented by lines with the indication of the mutation from the immediate ancestral haplotype (*kdr mutations in bold*). Grey circles: wild-type L1014 allele. Red circles: mutant L1014F allele (*G-5-T* or *G-5-C*). Yellow circles: mutant L1014C allele (*T-4-G*). Green circles: mutant L1014S allele (*T-4-C*). The numbers from 1 to 8 on the connection lines represent the nucleotide positions at 181, 940, 943, 1161, 1162,1197, 1210 and 1226 when compared with a template sequence (GenBank KP763726)

Among the 65 polymorphic sites detected within intron-1 and intron-2, six (at nucleotide positons: 181, 940, 943, 1197, 1210, 1226 when compared with a template sequence (GenBank KP763726)) showed to be highly polymorphic (>10 %; three sites from intron-1 and three from intron-2), and a total of 18 haplotypes were identified based on these six polymorphic sites (Additional file 1: Table S2). These six polymorphic sites were informative and used with two variable sites of *kdr* codon L1014 (at nucleotide positons: 1161 and 1162) for constructing a TCS genealogical network. The network revealed at least eight independent mutation events from seven distinct intron haplotypes, giving rise to nine *kdr* haplotypes (Fig. 2). Haplotypes H1-L1014F and H1-L1014C consist of the majority of the samples from central China and the two largest mutation groups, which are derived from the ancestor H1-L1014 with a single mutational step and two mutational steps, respectively. Haplotypes H07-L1014S and H08-L1014S from south China (Guangdong province) are derived from single mutational steps from two distinct ancestors H07-L1014 and H08-L1014, respectively, but with connections between these *kdr* haplotypes. Similarly, Haplotypes H04-L1014F and H14-L1014F are results of single mutational steps from two distinct ancestors H04-L1014 and H14-L1014, respectively, but with connection (*A-1-G*) between them. Haplotypes H04-L1014F (*kdr* L1014F codon TTT) and H04-L1014F (*kdr* L1014F codon TTC) are results of single mutational steps from the common ancestor H04-L1014, but with connection (*T-5-C*) between these *kdr* haplotypes. The remaining two *kdr* haplotypes (H05-L1014F and H02-L1014F) are also derived from single mutational steps from the distinct ancestors with a mixed geographical distribution from south to north.

### Mitochondrial DNA sequence variation

A total of 341 sequences for COI and 303 sequences for COII were generated for the 15 populations (Additional file 1: Table S3). COI and COII sequences were aligned giving a total length of 814 and 704 bp, respectively. The COI sequence alignment showed a total of 146 variable sites, of these 78 were parsimony informative. The COII sequence alignment revealed 85 variable sites, including 41 parsimony informative. Nucleotide diversity, number of haplotypes and haplotype diversity were higher in COI (*p* = 0.88 × 10^−2^; h = 238; *H*d = 0.99; Additional file 1: Table S3) than in COII (*p* = 0.47 × 10^−2^; h = 142; *H*d = 0.93). Among the 15 populations, *An. sinensis* from southern China exhibited higher haplotype and nucleotide diversity in COI and COII than those in central China (Additional file 1: Table S3).

### Genetic differentiation among populations

AMOVA found a significant population structure in the 15 *An. sinensis* populations examined, based on the combined COI and COII sequences (*F*_ST_ = 0.027, *P* < 0.0001). Population differentiation was significant between the southwest and south/central China regions (*F*_ST_ = 0.069, *P* < 0.001). Both the *F*_ST_ and *Ф*_ST_ values (based only on haplotype frequencies) were significant for 30 and 26 out of the 105 pairwise comparisons, respectively, after Bonferroni correction for multiple comparisons (Additional file 1: Table S4). The majority of the differences were detected between the two Yunnan populations (YNLH and YNNE) and the remaining populations.

### Spatial genetic structure analysis

**Fig. 3:**
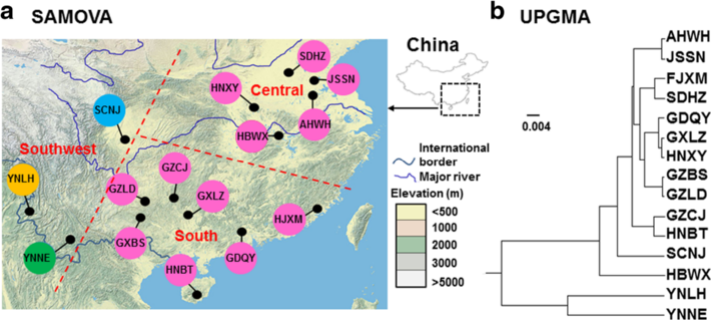
Cluster analysis based on the combined COI and COII sequences in *Anopheles sinensis* populations. **a** Colour codes of populations correspond to the five groups defined by SAMOVA; **b** UPGMA dendogram based on Nei‘s unbiased genetic distance between the 15 populations of *An. sinensis*

SAMOVA analyses with combined COI and COII sequences revealed a distinct decrease in *F*_CT_ value from K = 2 to 3, and *F*_CT_ values continue to decrease from K = 4 to 10. Although the *F*_CT_ value was highest at K = 2, the major decrease on *F*_CT_ occurred from K = 2 to 3 and from K = 3 to 4, with values only decreasing slightly thereafter. Similar patterns were also evident on *F*_ST_ and *F*_SC_ values (Additional file 1: Table S5). When K ≥ 5, the *F*_SC_ value became negative suggesting that there is no genetic barrier between populations within groups (Fig. 3a). The two Yunnan populations (YNLH and YNNE) were assigned to two single population groups when K = 3 with the highest *F*_CT_ values, suggesting that there is genetic barrier to restrict gene flow from Yunnan to the other populations examined. In general, the patterns found in SAMOVA analyses were consistent with the phylogenetic trees of populations (Fig. 3b). The UPGMA dendogram based on Nei‘s unbiased genetic distances between populations showed two distinctive groups in which the two populations, YNLH and YNNE from Yunnan province constituted a cluster and the remaining populations from south and central China were included in a second cluster (Fig. 3b).

**Fig. 4:**
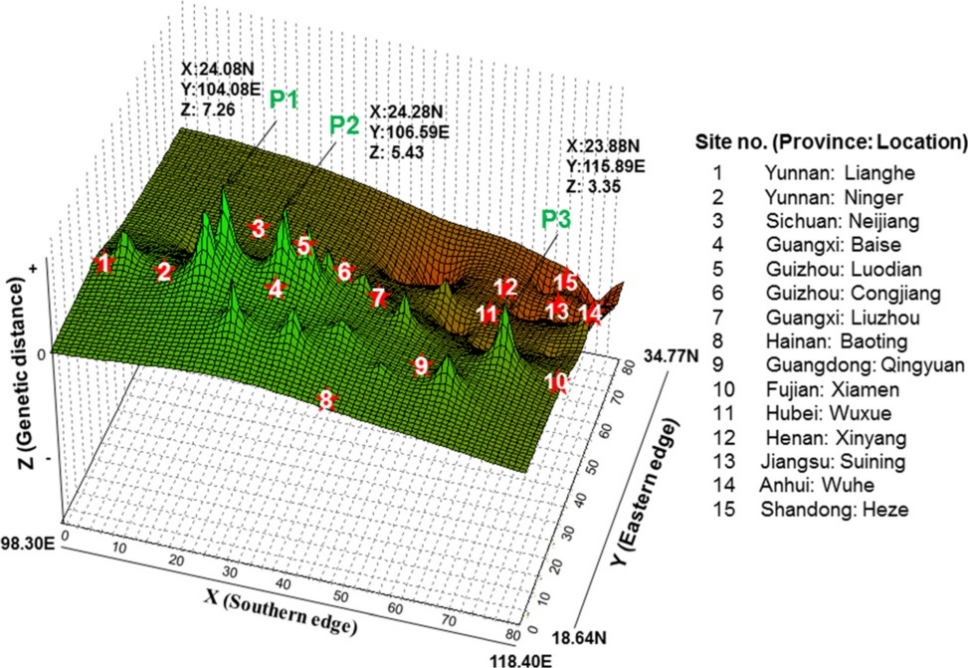
Genetic landscape shape plot showing patterns of spatial genetic distance for 15 populations of *Anopheles sinensis*. Geographical coordinates are shown in the X (North–south) and Y (East–west) axes. Z axis (height) corresponds to the genetic distance between individuals. Green peaks (P1, P2 and P3) are indicative of areas with high pairwise genetic distances and red valleys are indicative of areas of low pairwise genetic distance

A Mantel test with combined COI and COII sequences revealed a significant correlation between geographical and genetic distances with all populations included (*R*^*2*^ = 0.164, *P* < 0.01, *n* = 105), suggesting that the genetic structure observed in the *An. sinensis* populations could be partially explained by distance isolation (Additional file 2: Figure S1). The genetic landscape shape analysis identified major potential gene flow barriers (Fig. 4). The highest peak of differentiation (P1) was found in southwest China between Yunnan and Guangxi/Sichuan populations, followed by the second highest peak (P2) in southern China between Sichuan and Guangxi/Guizhou/Guangdong. A third peak (P3) was identified between the populations from the south and central China. Low genetic differentiation was detected between populations in the central China region, as shown by the red valleys (Fig. 4).

### Demographic history and neutrality test based on mtDNA sequences

Tajima’s *D* and Fu’s *F*s tests found that 5 and 13 of the 15 populations were significantly negative, suggesting a recent population expansion or selection (Table 3). R_2_ statistics found similar results with Fu’s *F*s tests. The two populations from Yunnan (YNLH and YNNE) showed nonsignificant *D*- and *F-*values, indicating an excess of rare nucleotide site variants compared to what would be expected under a neutral model of evolution. The observed smooth and unimodal mismatch distribution indicates sudden population expansion in 13 out of the 15 populations, and this was consistent with the mismatch distribution derived under the model of sudden expansion (Additional file 2: Figure S2). On the other hand, the multimodal pattern of mismatch distributions observed in the Yunnan populations suggested that no expansion events were detected and population size remained constant over time (Additional file 2: Figure S2).

**Table 3:** Analysis of historical demography. Variation of estimated demographic parameters based on mitochondrial COI and COII sequence data of 15 populations of *An. sinensis*

Population	N	Tajima’s *D*	Fu’s *Fs*	SSD	*r*	R2	τ	Time (ky)
Southwest								
YNLH	22	−0.51	−3.69	0.018	0.022	0.1014	5.027	144
YNNE	21	0.17	−1.28	0.016	0.031	0.1325	5.178	148
SCNJ	20	−1.39	−9.27**	0.004	0.008	0.0717**	5.392	154
South								
GXBS	21	−1.56*	−9.83**	0.007	0.015	0.0719*	7.85	225
GZLD	20	−1.1	−8.24**	0.005	0.009	0.0827*	5.622	161
GZCJ	18	−1.45	−9.65**	0.077	0.01	0.0683**	5.877	168
GXLZ	18	−1.28	−11.78**	0.011	0.015	0.0764*	6.232	178
HNBT	12	−1.07	−4.53*	0.005	0.012	0.0872**	8.249	236
GDQY	19	−1.17	−7.90**	0.007	0.01	0.0838*	7.452	213
FJXM	21	−1.4	−7.03**	0.012	0.023	0.0663**	6.844	196
Central								
HBWX	20	−1.67*	−11.93**	0.007	0.019	0.0564**	6.03	173
HNXY	21	−1.67*	−13.59**	0.004	0.007	0.0652**	5.726	164
JSSN	24	−1.46	−18.64**	0.004	0.008	0.0613**	6.477	185
AHWH	17	−1.62*	−9.60**	0.037	0.015	0.0672**	6.113	175
SDHZ	18	−1.83*	−11.96**	0.003	0.005	0.0621**	4.841	139

*N* number of samples analysed, *SSD* the sum of squared deviations, *R2* Ramos-Onsins & Rozas’s R2, *r* Harpending’s raggedness index, *τ* estimated parameter of population expansion assuming the stepwise growth model (*τ* = 2 t*MU where t = time in years, MU = mutation rate per locus)

*P < 0.05; **P < 0.01 (FDR < 0.02)

### Migration and gene flow patterns

**Fig. 5:**
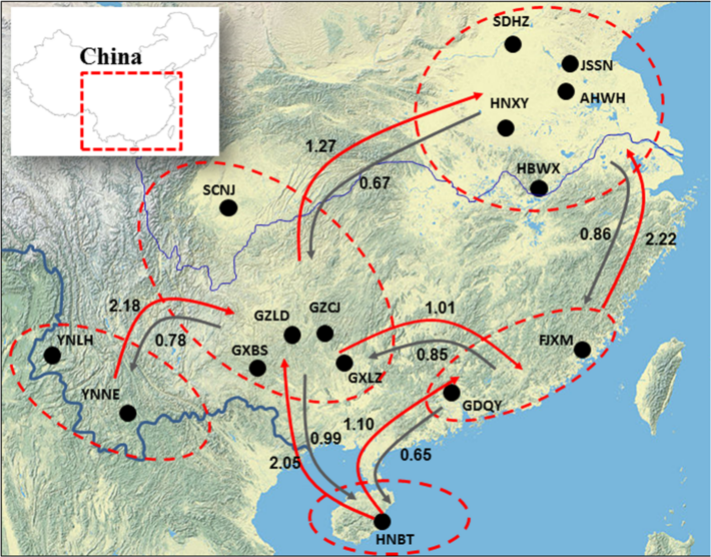
Bayesian estimates of historical asymmetrical migration between populations of *Anopheles sinensis*. The five locality groups are indicated by dotted circles and arrows indicate the direction of migration rates

LAMARC analysis found historical gene flow rates ranged from 0.01 to 4.85 (Additional file 1: Table S6). The highest migration rates were detected among neighboring populations within each of the five locality groups (Fig. 5). Among the five locality groups, medium levels of migration were observed. The Hainan (HNBT) and Yunnan (YNLH and YNNE) populations were apparently isolated with little gene flow (0.01 < Nem < 0.85). Based on the coalescent analysis, migration was asymmetrical, i.e., higher from the south to the north, and from southwest to the central regions than the opposite directions (Fig. 5).

## Discussion

Near fixation of *kdr* mutation was detected in populations from central China, but no *kdr* mutations were found in populations from Yunnan and Sichuan provinces. Absence of *kdr* mutations in *An. sinensis* samples from southwestern China and low frequency of *kdr* mutations in southern China is likely a consequence of geographical isolation in the mosquito populations combined with the absence of strong local selection.

### Geographical isolation of *An. sinensis* populations from southwestern China

Based on mtDNA sequencing, significant genetic differentiation was detected between Yunnan (southwestern China) and populations from south/central China. The AMOVA analysis indicated that the percentage of genetic variation between these two regions (~28 %) is much higher than that among populations within each of the regions (~3 %). Such regional genetic differentiation was also manifested by the observation of more private alleles in each of the regions than within individual populations of a region. Our data show that *An. sinensis* in Yunnan has a relatively stable population size over time without drastic demographic changes, unlike populations from central and southern China. The strong genetic differentiation and limited gene flow between the Yunnan and central China populations suggest that these two regions are genetically isolated. SAMOVA analysis further confirmed that there is a genetic barrier restricting gene flow between the Yunnan populations and the other populations examined. These patterns were also consistent with the phylogenetic trees of populations. The two Yunnan populations are located in the Yunnan Plateau, a mountainous area in southwest China with high elevations in the northwest and low elevations in the southeast of Yunnan province. Therefore, both physical distance and heterogeneous landscape could be factors inhibiting gene flow between Yunnan and south/central China. However, based on the analysis of DNA sequences surrounding *kdr* codon L1014, significant genetic differentiation was detected between the central China populations and the remaining populations, suggesting a strong local selection of the *kdr* resistance allele in the central China populations.

### Effects of insecticide-associated selection

For the two Yunnan populations, neutrality tests (Fu’s *F*s statistic) did not provide evidence of selection at the *kdr* introns. This finding concurs with a relatively low rate of insecticide use in agriculture and indoor spraying in Yunnan as compared to central China. Apart from limited migrations, the absence of strong selective pressure may in part explain the lack of insecticide-resistance alleles in Yunnan. Lower genetic variation was observed in the introns of the *kdr* locus in populations from central China when compared to the other populations. These populations showed a high frequency of *kdr* mutation (L1014F), suggesting that low variation at the adjacent introns could be a consequence of a recent selective sweep associated with intense insecticide use following the selection and/or fixation of the resistance genotypes. A selective sweep occurs when an allele rapidly increases its frequency due to positive or directional selection. Through genetic hitchhiking, the frequency of linked alleles in the flanking regions of the locus under selection can also increase, thus reducing genetic variation [65, 66].

It must be noted that among the different neutrality tests, Fu’ *F*_S_ statistic shows the greatest power in detecting departures from neutrality under a genetic hitchhiking model [46]. The high Fu’s *F*_S_ estimates for central China could reflect the impact of strong selection by the increased use of insecticide not only on the *kdr* locus, but also on the other gene regions. In the past half-century, insecticides are known to have been extensively used for agricultural and vector control purposes in central China [33]. Rice is the major agricultural crop in these study sites, with 1–2 harvests per year. Due to severe insect pest damage, insecticide use for pest control has been very intensive, with several rounds of sprays administered during each growing season. Since the mid-1980s, pyrethroids have been the dominant insecticides with pyrethroids-treated areas constituting more than one third of the total insecticide-treated areas in central China [37, 67, 68]. In addition to their agricultural use, pyrethroids have had various public health applications, e.g., as indoor sprays or incense, impregnated in bed nets, or as tools in public sanitation [25, 37, 67, 68]. The large scale agricultural use and indoor residual spraying of pyrethroid insecticides have probably generated selective pressure at the sodium channel gene in local *An. sinensis* populations. *Anopheles sinensis* from central China were found with extremely high resistant to pyrethroids, organochlorine, organophosphates and carbamates [31, 33]. Thus, it is not surprising for selection to have operated beyond the upstream and downstream introns of the *kdr* locus and towards parts of the mosquito’s genome in central China.

### Multiple origins of *kdr* mutations

Information on whether resistance-associated mutations evolved independently *vs* single emergency and then spatially spread is valuable to elucidate the relative importance of mutation and migration in the spread of insecticide resistance. We detected three non-synonymous mutations at codon L1014 of the sodium channel gene. The majority of samples from central China harbored the *kdr* mutation L1014F, whereas the L1014S mutation was only found in Guangdong Province, southern China. The L1014C mutation was detected in both southern and central China. This result is consistent with previous study [37] of *kdr* genotyping in *An. sinensis* which found that the L1014F mutation was largely distributed in central China. Analysis of an upstream intron (intron-1) and a downstream intron (intron-2) of the *kdr* codon suggests at least eight independent origins of *kdr* alleles in *An. sinensis* populations from south to central China. Multiple origins of *kdr* alleles were also reported in the Afrotropical mosquito vector *An. gambiae* [1]. The *kdr* haplotypes H01-1014 F and H01-L1014C from the same ancestor were the most widespread in *An. sinensis* populations from central China and evolved multiple times subsequent to their first divergence. This phenomenon could be explained by the selection pressure of insecticides experienced in these populations, which favors *kdr* resistant alleles. Therefore, the frequency of haplotype (H01-L1014) carrying the wild-type allele is much lower than that of haplotype (H01-L1014F) harbouring *kdr* mutations. However, the *kdr* haplotype L1014S from two distinct ancestors was detected only in the Guangdong population, suggesting a recent origin of this mutation that is unique to the populations in southern China.

## Conclusions

Our results indicate multiple origins of the *kdr* insecticide-resistant alleles in *An. sinensis* from southern and central China. Local selection related to intense and prolonged use of insecticide for agricultural purposes, as well as frequent migrations among populations are likely the explanations for the patchy distributions of *kdr* mutations in China. On the contrary, the lack of *kdr* mutations in Yunnan and Sichuan is likely a consequence of genetic isolation and absence of strong selection pressure. The present study compares the genetic patterns revealed by a functional gene with a neutral marker and demonstrates the combined impact of demographic and selection factors on population structure.

## Additional files

Additional file 1: Table S1.
*kdr* L1014 codon region DNA based population differentiation for population pairs (estimates of *F*_ST_ below diagonal and *Ф*_ST_ above diagonal). **Table S2.** Distribution of Intron-1 and intron-2 haplotypes of *kdr* allele in the 15 *Anopheles sinensis* populations. **Table S3.** Haplotype and nucleotide diversity of the mitochondrial cytochrome *c* oxidase gene. **Table S4.** Mitochondrial DNA based population differentiation for population pairs (estimates of *F*_ST_ below diagonal and *Ф*_ST_ above diagonal). **Table S5.** List of grouping results from the spatial analysis of molecular variance (SAMOVA) showing values for variation among groups (*F*_CT_), among populations among groups (*F*_ST_) and among populations within groups (*F*_SC_). **Table S6.** Estimates of gene flow (migration rate) among the 15 *Anopheles sinensis* populations in China. (DOCX 44 kb) Additional file 2: Figure S1. Isolation by distance, relationship between geographical and genetic distances based on mtDNA sequences in the *Anopheles sinensis* populations. **a**, All populations; **b**, All populations without Yunnan samples. **Figure S2.** Observed (Obs) and expected (Exp) mismatch distributions showing the frequencies of pairwise differences. The observed distributions (red dot lines) are compared for their goodness-of-fit to a Poisson distribution under a model of sudden expansion illustrated by the overlaid curve (green solid lines). **a**, All populations; **b**, All populations without Yunnan; and **c**, Two populations from Yunnan province. (PDF 336 kb)
