# Supplementary material for: Landscape genetic structure and evolutionary genetics of insecticide resistance gene mutations in Anopheles sinensis
Source: Parasit Vectors. 2016 Apr 23;9:228. doi: 10.1186/s13071-016-1513-6 (PMC4842280; doi:10.1186/s13071-016-1513-6)
Supplement: Additional file 1: Table S1. — kdr L1014 codon region DNA based population differentiation for population pairs (estimates of F ST below diagonal and Ф ST above diagonal). Table S2. Distribution of Intron-1 and intron-2 haplotypes of kdr allele in the 15 Anopheles sinensis populations. Table S3. Haplotype and nucleotide diversity of the mitochondrial cytochrome c oxidase gene. Table S4. Mitochondrial DNA based population differentiation for population pairs (estimates of F ST below diagonal and Ф ST above diagonal). Table S5. List of grouping results from the spatial analysis of molecular variance (SAMOVA) showing values for variation among groups (F CT), among populations among groups (F ST) and among populations within groups (F SC). Table S6. Estimates of gene flow (migration rate) among the 15 Anopheles sinensis populations in China. (DOCX 44 kb) [file 13071_2016_1513_MOESM1_ESM.docx]

**Supplementary Table S1** *kdr* L1014 codon region DNA based population differentiation for population pairs (estimates of *F*_ST_ below diagonal and *Ф*_ST_ above diagonal).

|  | YNLH | YNNE | SCNJ | GXBS | GZLD | GZCJ | GXLZ | HNBT | GDQY | FJXM | HBWX | HNXY | JSSN | AHWH | SDHZ |
| --- | --- | --- | --- | --- | --- | --- | --- | --- | --- | --- | --- | --- | --- | --- | --- |
| YNLH | - | -0.024 | 0.007 | 0.033 | 0.019 | -0.012 | 0.104 | 0.033 | 0.035 | 0.063 | ***0.482*** | ***0.526*** | ***0.525*** | ***0.576*** | ***0.390*** |
| YNNE | -0.018 | - | 0.009 | 0.035 | 0.017 | -0.014 | 0.083 | 0.024 | 0.036 | 0.044 | ***0.475*** | ***0.522*** | ***0.523*** | ***0.577*** | ***0.377*** |
| SCNJ | -0.010 | -0.018 | - | 0.054 | 0.008 | -0.003 | 0.068 | 0.044 | 0.060 | 0.022 | ***0.434*** | ***0.482*** | ***0.478*** | ***0.533*** | ***0.334*** |
| GXBS | -0.005 | 0.004 | 0.015 | - | 0.053 | 0.007 | ***0.198*** | 0.003 | 0.035 | 0.036 | ***0.348*** | ***0.269*** | ***0.378*** | ***0.340*** | ***0.232*** |
| GZLD | ***0.184*** | ***0.206*** | ***0.125*** | ***0.149*** | - | 0.091 | 0.184 | 0.083 | 0.205 | 0.140 | ***0.468*** | ***0.400*** | ***0.507*** | ***0.456*** | ***0.349*** |
| GZCJ | 0.025 | 0.043 | 0.008 | 0.002 | 0.003 | - | 0.055 | 0.012 | 0.036 | 0.047 | ***0.307*** | ***0.243*** | ***0.333*** | ***0.302*** | ***0.219*** |
| GXLZ | 0.047 | 0.030 | 0.045 | 0.042 | 0.077 | 0.083 | - | 0.156 | 0.081 | 0.008 | ***0.236*** | ***0.134*** | ***0.261*** | ***0.214*** | ***0.153*** |
| HNBT | 0.066 | 0.067 | 0.070 | 0.052 | ***0.188*** | 0.060 | 0.044 | - | 0.088 | 0.063 | ***0.348*** | ***0.270*** | ***0.375*** | ***0.340*** | ***0.239*** |
| GDQY | 0.038 | 0.053 | 0.054 | 0.075 | 0.030 | 0.016 | 0.094 | 0.083 | - | 0.086 | ***0.363*** | ***0.288*** | ***0.388*** | ***0.354*** | ***0.262*** |
| FJXM | 0.045 | 0.034 | 0.035 | ***0.169*** | 0.028 | 0.054 | -0.005 | ***0.145*** | 0.087 | - | ***0.238*** | ***0.186*** | ***0.269*** | ***0.240*** | ***0.153*** |
| HBWX | ***0.342*** | ***0.338*** | ***0.343*** | ***0.597*** | ***0.443*** | ***0.442*** | ***0.208*** | ***0.538*** | ***0.435*** | ***0.308*** | - | 0.027 | -0.016 | 0.001 | ***0.172*** |
| HNXY | ***0.274*** | ***0.265*** | ***0.279*** | ***0.646*** | ***0.499*** | ***0.489*** | ***0.246*** | ***0.582*** | ***0.474*** | ***0.374*** | 0.059 | - | 0.014 | 0.009 | ***0.228*** |
| JSSN | ***0.366*** | ***0.364*** | ***0.369*** | ***0.655*** | ***0.495*** | ***0.484*** | ***0.252*** | ***0.584*** | ***0.474*** | ***0.365*** | -0.022 | 0.066 | - | -0.002 | ***0.219*** |
| AHWH | ***0.337*** | ***0.333*** | ***0.340*** | ***0.693*** | ***0.548*** | ***0.541*** | ***0.306*** | ***0.635*** | ***0.529*** | ***0.428*** | 0.010 | -0.006 | -0.012 | - | ***0.253*** |
| SDHZ | ***0.242*** | ***0.231*** | ***0.240*** | ***0.537*** | ***0.344*** | ***0.366*** | ***0.141*** | ***0.489*** | ***0.368*** | ***0.178*** | ***0.186*** | ***0.163*** | ***0.211*** | ***0.185*** | - |

*F*_ST_ values based only on haplotype frequencies

*Ф*_ST_ estimates based on both haplotype frequencies and a measure of genetic distance

Value in bold italic indicates significant at *P* < 0.01 after Bonferroni correction

**Supplementary Table S2** Distribution of Intron-1 and intron-2 haplotypes of *kdr* allele in the 15 *Anopheles sinensis* populations.

| Haplotype | YNLH | YNNE | SCNJ | GXBS | GZLD | GZCJ | GXLZ | HNBT | GDQY | FJXM | HBWX | HNXY | JSSN | AHWH | SDHZ | *kdr* allele* |
| --- | --- | --- | --- | --- | --- | --- | --- | --- | --- | --- | --- | --- | --- | --- | --- | --- |
| Hap_1 | 2 | 2 | 7 | 1 | 16 | 8 | 13 | 4 | 1 | 10 | 36 | 33 | 27 | 55 | 21 | C49F139L48 |
| Hap_2 | 4 | 4 | 9 |  |  | 3 | 3 |  |  | 1 | 2 | 4 | 2 | 5 |  | F14L23 |
| Hap_3 |  |  | 1 |  |  |  | 1 |  |  |  |  |  |  | 1 |  | C2L1 |
| Hap_4 |  |  | 2 | 2 |  | 2 | 2 | 1 |  | 1 | 4 |  | 1 | 1 | 1 | F7L10 |
| Hap_5 | 12 | 9 | 10 | 4 |  | 7 | 5 | 5 | 17 | 4 |  |  |  |  |  | F7L66 |
| Hap_6 | 1 |  |  |  |  |  |  | 1 | 1 | 3 |  |  |  |  |  | L6 |
| Hap_7 | 9 | 4 | 11 | 6 | 15 | 15 | 1 | 5 | 11 | 2 |  |  |  |  |  | S1L78 |
| Hap_8 | 8 | 6 | 4 | 4 | 1 | 2 | 2 | 2 | 5 | 2 |  |  |  |  |  | S5L31 |
| Hap_9 |  |  |  |  |  |  |  |  |  | 1 |  |  |  |  |  | L1 |
| Hap_10 |  | 1 |  | 1 |  |  | 1 |  |  | 2 |  |  |  |  |  | L5 |
| Hap_11 | 2 | 1 |  | 1 |  |  |  |  | 1 |  |  |  |  |  |  | L5 |
| Hap_12 |  |  |  | 2 |  |  |  |  |  |  |  |  |  |  |  | L2 |
| Hap_13 |  | 2 |  | 1 |  |  |  |  |  |  |  |  |  |  |  | L3 |
| Hap_14 |  |  |  |  |  | 1 | 2 | 11 |  |  |  |  |  |  |  | F1L13 |
| Hap_15 | 3 | 3 |  |  |  | 1 | 1 |  |  |  |  | 1 |  |  |  | L9 |
| Hap_16 |  |  |  |  |  | 1 | 1 |  |  |  |  |  |  |  |  | L2 |
| Hap_17 | 1 |  |  |  |  |  |  | 1 |  |  |  |  |  |  |  | L2 |
| Hap_18 |  |  |  |  |  |  |  |  |  |  |  |  |  |  | 16 | L16 |

* L=L1014, F=L1014F, C=1014C, S=1014S. Number following allele means the number of sequences.

**Supplementary Table S3** Haplotype and nucleotide diversity of the mitochondrial cytochrome c oxidase gene.

| Pop &Gene | Sample size | # of variable sites | # of parsimony informative sites | # of haplotypes  h | Haplotype diversity, *H*d | Nucleotide diversity (p×100) |
| --- | --- | --- | --- | --- | --- | --- |
| COI gene | |  |  |  |  |  |
| YNLH | 26 | 33 | 19 | 17 | 0.90 | 0.95 |
| YNNE | 24 | 21 | 17 | 11 | 0.87 | 0.79 |
| SCNJ | 22 | 36 | 16 | 19 | 0.97 | 0.77 |
| GXBS | 27 | 41 | 22 | 26 | 1.00 | 0.97 |
| GZLD | 20 | 37 | 19 | 19 | 0.99 | 0.93 |
| GZCJ | 27 | 46 | 22 | 26 | 1.00 | 0.96 |
| GXLZ | 21 | 40 | 14 | 19 | 0.99 | 0.87 |
| HNBT | 12 | 31 | 13 | 12 | 1.00 | 1.00 |
| GDQY | 20 | 35 | 16 | 16 | 0.96 | 0.88 |
| FJXM | 22 | 46 | 16 | 19 | 0.98 | 0.96 |
| HBWX | 21 | 30 | 10 | 17 | 0.97 | 0.61 |
| HNXY | 22 | 42 | 15 | 21 | 0.99 | 0.89 |
| JSSN | 32 | 46 | 20 | 29 | 0.99 | 0.81 |
| AHWH | 22 | 40 | 15 | 21 | 1.00 | 0.78 |
| SDHZ | 23 | 37 | 12 | 22 | 1.00 | 0.66 |
|  |  |  |  |  |  |  |
| COII gene | |  |  |  |  |  |
| YNLH | 23 | 21 | 9 | 14 | 0.90 | 0.54 |
| YNNE | 22 | 17 | 8 | 12 | 0.90 | 0.55 |
| SCNJ | 20 | 11 | 4 | 10 | 0.84 | 0.24 |
| GXBS | 22 | 25 | 13 | 22 | 1.00 | 0.65 |
| GZLD | 21 | 21 | 13 | 13 | 0.91 | 0.60 |
| GZCJ | 18 | 19 | 8 | 14 | 0.97 | 0.50 |
| GXLZ | 18 | 22 | 6 | 17 | 0.99 | 0.52 |
| HNBT | 12 | 13 | 3 | 9 | 0.94 | 0.41 |
| GDQY | 19 | 19 | 8 | 12 | 0.92 | 0.50 |
| FJXM | 22 | 19 | 10 | 14 | 0.92 | 0.49 |
| HBWX | 23 | 19 | 7 | 16 | 0.91 | 0.4 |
| HNXY | 21 | 21 | 5 | 16 | 0.96 | 0.42 |
| JSSN | 24 | 14 | 6 | 14 | 0.90 | 0.29 |
| AHWH | 20 | 18 | 10 | 14 | 0.92 | 0.46 |
| SDHZ | 18 | 18 | 4 | 11 | 0.90 | 0.41 |

**Supplementary Table S4** Mitochondrial DNA based population differentiation for population pairs (estimates of *F*_ST_ below diagonal and *Ф*_ST_ above diagonal).

|  | YNLH | YNNE | SCNJ | GXBS | GZLD | GZCJ | GXLZ | HNBT | GDQY | FJXM | HBWX | HNXY | JSSN | AHWH | SDHZ |
| --- | --- | --- | --- | --- | --- | --- | --- | --- | --- | --- | --- | --- | --- | --- | --- |
| YNLH | - | 0.045 | ***0.066*** | 0.026 | 0.026 | 0.040 | ***0.045*** | 0.021 | 0.039 | ***0.044*** | ***0.115*** | ***0.058*** | ***0.071*** | ***0.046*** | ***0.077*** |
| YNNE | 0.003 | - | ***0.120*** | ***0.053*** | ***0.078*** | ***0.092*** | ***0.083*** | ***0.064*** | ***0.099*** | ***0.081*** | ***0.179*** | ***0.084*** | ***0.107*** | ***0.104*** | ***0.122*** |
| SCNJ | ***0.032*** | ***0.039*** | - | ***0.036*** | 0.025 | 0.018 | -0.001 | 0.031 | 0.022 | ***0.032*** | ***0.034*** | 0.020 | 0.031 | 0.006 | ***0.025*** |
| GXBS | ***0.024*** | ***0.031*** | 0.008 | - | -0.001 | 0.005 | -0.001 | -0.029 | 0.003 | -0.008 | ***0.062*** | -0.010 | 0.001 | 0.002 | ***0.025*** |
| GZLD | ***0.027*** | ***0.034*** | 0.011 | 0.003 | - | -0.026 | -0.011 | -0.010 | -0.019 | 0.007 | 0.025 | 0.003 | 0.028 | -0.021 | 0.004 |
| GZCJ | ***0.024*** | ***0.029*** | 0.008 | -0.003 | -0.003 | - | -0.013 | -0.004 | -0.011 | -0.004 | 0.008 | -0.011 | 0.022 | -0.019 | -0.007 |
| GXLZ | ***0.024*** | ***0.029*** | 0.008 | 0.000 | -0.003 | -0.006 | - | -0.006 | 0.001 | -0.003 | 0.007 | 0.000 | 0.016 | -0.011 | -0.002 |
| HNBT | 0.017 | 0.025 | 0.008 | 0.000 | -0.006 | -0.005 | -0.005 | - | -0.025 | -0.011 | ***0.057*** | -0.008 | -0.003 | -0.006 | 0.023 |
| GDQY | ***0.027*** | ***0.034*** | 0.011 | 0.003 | 0.000 | 0.003 | 0.003 | -0.001 | - | 0.015 | 0.039 | 0.005 | 0.015 | -0.011 | 0.021 |
| FJXM | ***0.029*** | ***0.036*** | ***0.013*** | 0.005 | 0.007 | 0.005 | 0.005 | 0.005 | 0.008 | - | ***0.042*** | -0.012 | 0.008 | 0.003 | 0.005 |
| HBWX | ***0.027*** | ***0.034*** | 0.011 | 0.003 | -0.005 | -0.003 | -0.006 | -0.006 | 0.006 | 0.007 | - | 0.022 | ***0.041*** | 0.003 | 0.006 |
| HNXY | ***0.024*** | ***0.031*** | 0.008 | 0.000 | 0.003 | 0.000 | 0.000 | 0.000 | 0.003 | 0.003 | 0.003 | - | -0.010 | -0.013 | -0.009 |
| JSSN | ***0.024*** | ***0.031*** | 0.008 | 0.000 | -0.002 | -0.002 | -0.002 | -0.003 | 0.003 | ***0.005*** | -0.002 | -0.002 | - | 0.000 | 0.025 |
| AHWH | ***0.024*** | ***0.031*** | 0.008 | 0.000 | 0.003 | 0.000 | 0.000 | 0.000 | 0.003 | 0.005 | 0.003 | 0.000 | 0.000 | - | -0.021 |
| SDHZ | ***0.024*** | ***0.031*** | 0.008 | 0.000 | -0.003 | -0.003 | -0.003 | -0.005 | 0.003 | 0.005 | -0.003 | -0.008 | -0.005 | 0.000 | - |

*F*_ST_ values based only on haplotype frequencies

*Ф*_ST_ estimates based on both haplotype frequencies and a measure of genetic distance

Value in bold italic indicates significant at P < 0.05 after Bonferroni correction

**Supplementary Table S5** List of grouping results from the spatial analysis of molecular variance (SAMOVA) showing values for variation among groups (*F*_CT_), among populations among groups (*F*_ST_), and among populations within groups (*F*_SC_).

| **K** | **Groupings** | ***F*_CT_** | ***F*_ST_** | ***F*_SC_** |
| --- | --- | --- | --- | --- |
| **2** | [YNNE][AHWH,FJXM,GDQY,GXBS,GXLZ,GZCJ,GZLD,HBWX,HNBT,HNXY,JSSN,SCNJ,SDHZ,YNLH] | 0.0932 | 0.1041 | 0.0119 |
| **3** | [YNNE][YNLH][AHWH,FJXM,GDQY,GXBS,GXLZ,GZCJ,GZLD,HBWX,HNBT,HNXY,JSSN,SCNJ,SDHZ] | 0.0757 | 0.0793 | 0.0038 |
| **4** | [YNNE][YNLH][HBWX][AHWH,FJXM,GDQY,GXBS,GXLZ,GZCJ,GZLD,HNBT,HNXY,JSSN,SCNJ,SDHZ] | 0.0618 | 0.0626 | 0.0008 |
| **5** | [YNNE][YNLH][HBWX][SCNJ][AHWH,FJXM,GDQY,GXBS,GXLZ,GZCJ,GZLD,HNBT,HNXY,JSSN,SDHZ] | 0.0545 | 0.0527 | -0.0019 |
| **6** | [YNNE][YNLH][HBWX][SCNJ][JSSN][AHWH,FJXM,GDQY,GXBS,GXLZ,GZCJ,GZLD,HNBT,HNXY,SDHZ] | 0.0482 | 0.0445 | -0.0039 |
| **7** | [YNNE][YNLH][HBWX][SCNJ][JSSN][FJXM,GXBS,HNBT][AHWH,GDQY,GXLZ,GZCJ,GZLD,HNXY,SDHZ] | 0.0462 | 0.0336 | -0.0132 |
| **8** | [YNNE][YNLH][HBWX][SCNJ][JSSN,HNXY][FJXM][GXBS,HNBT][AHWH,GDQY,GXLZ,GZCJ,GZLD,SDHZ] | 0.0465 | 0.0299 | -0.0173 |
| **9** | [YNNE][YNLH][HBWX][SCNJ,GXLZ][JSSN,HNXY][FJXM][GXBS,HNBT][AHWH,SDHZ][GDQY,GZCJ,GZLD] | 0.0482 | 0.0292 | -0.0199 |
| **10** | [YNNE][YNLH][HBWX][SCNJ,GXLZ][JSSN,HNXY][FJXM][GXBS,HNBT][AHWH,SDHZ][GDQY][GZCJ,GZLD] | 0.0498 | 0.0284 | -0.0225 |
