# Supplementary material for: Landscape genetic structure and evolutionary genetics of insecticide resistance gene mutations in Anopheles sinensis
Source: Parasit Vectors. 2016 Apr 23;9:228. doi: 10.1186/s13071-016-1513-6 (PMC4842280; doi:10.1186/s13071-016-1513-6)
Supplement: Additional file 2: Figure S1. — Isolation by distance, relationship between geographical and genetic distances based on mtDNA sequences in the Anopheles sinensis populations. a, All populations; b, All populations without Yunnan samples. Figure S2. Observed (Obs) and expected (Exp) mismatch distributions showing the frequencies of pairwise differences. The observed distributions (red dot lines) are compared for their goodness-of-fit to a Poisson distribution under a model of sudden expansion illustrated by the overlaid curve (green solid lines). a, All populations; b, All populations without Yunnan; and c, Two populations from Yunnan province. (PDF 336 kb) [file 13071_2016_1513_MOESM2_ESM.pdf]

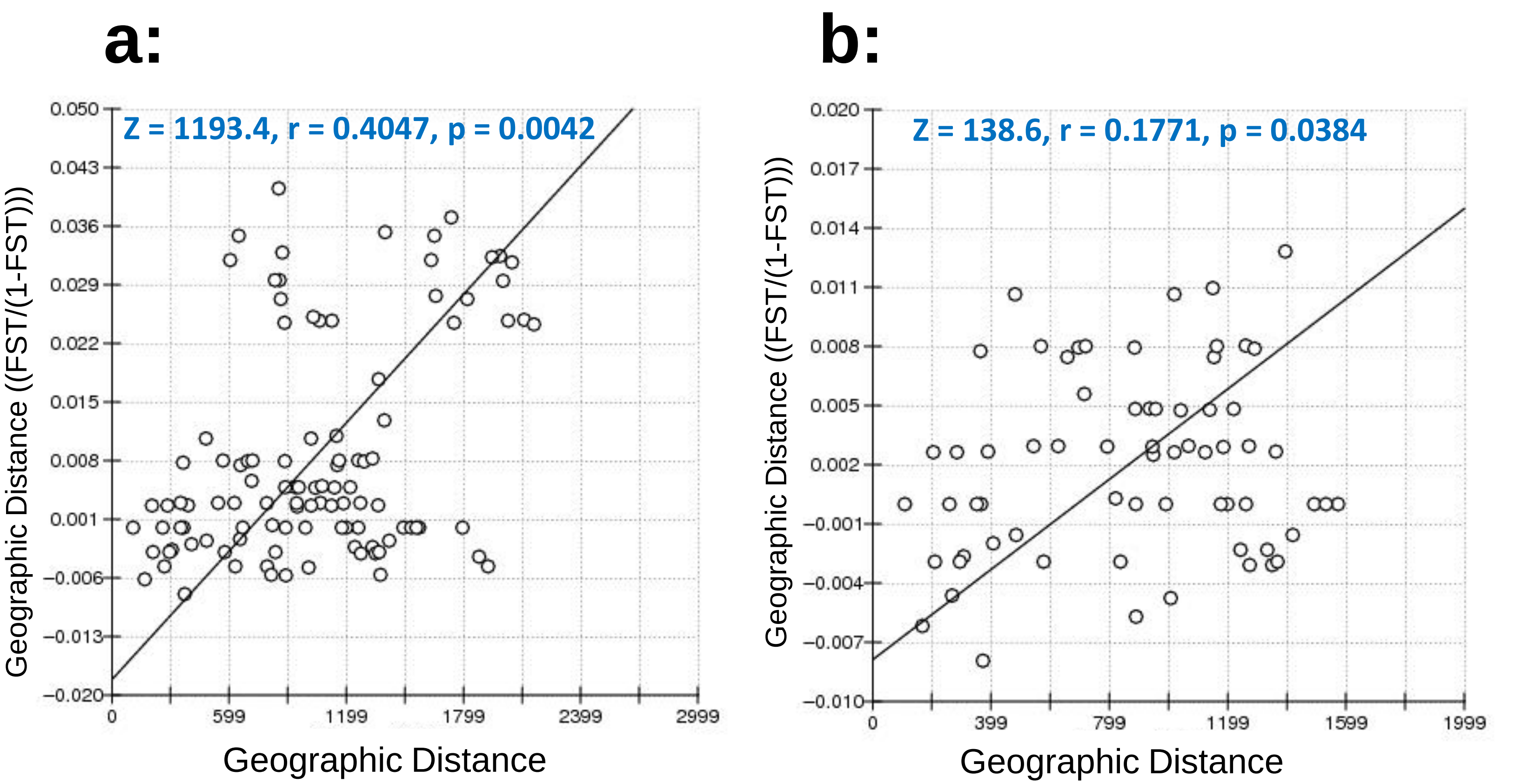

Additional file 2: Figure S1. Isolation by distance, relationship between geographical and genetic distances based on mtDNA sequences in the *Anopheles sinensis* populations. **a**, All populations; **b**, All populations without Yunnan samples.

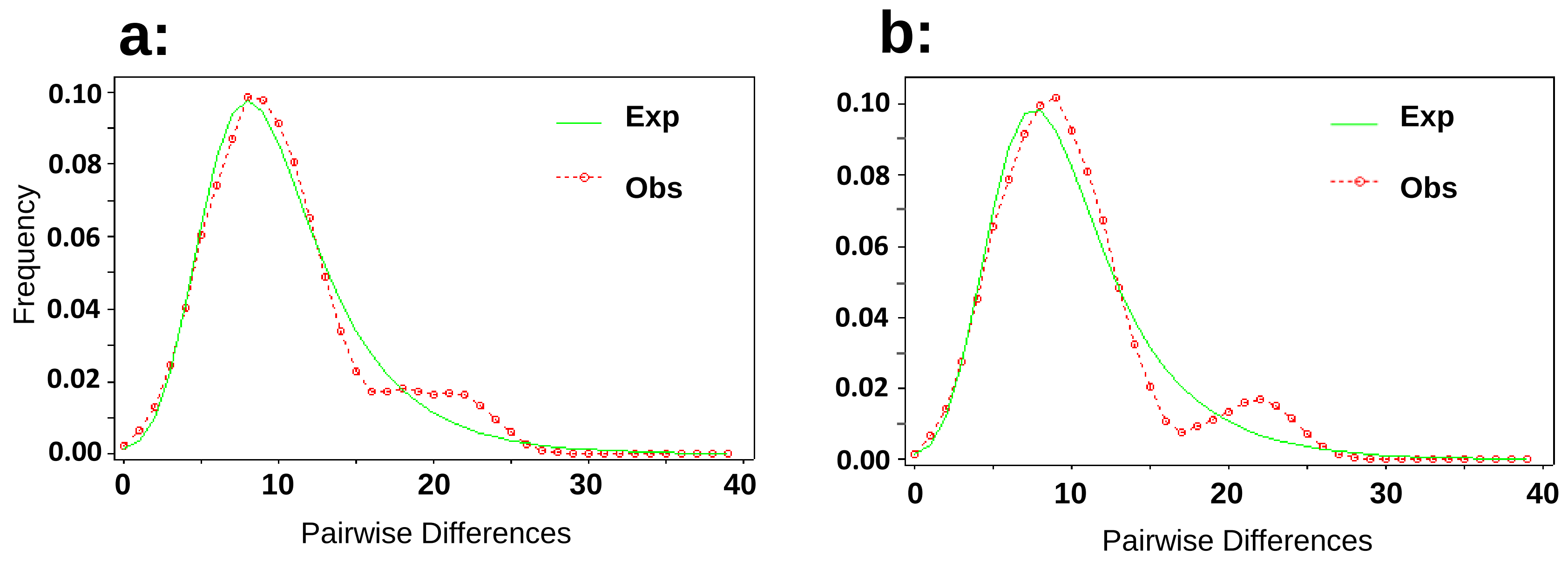

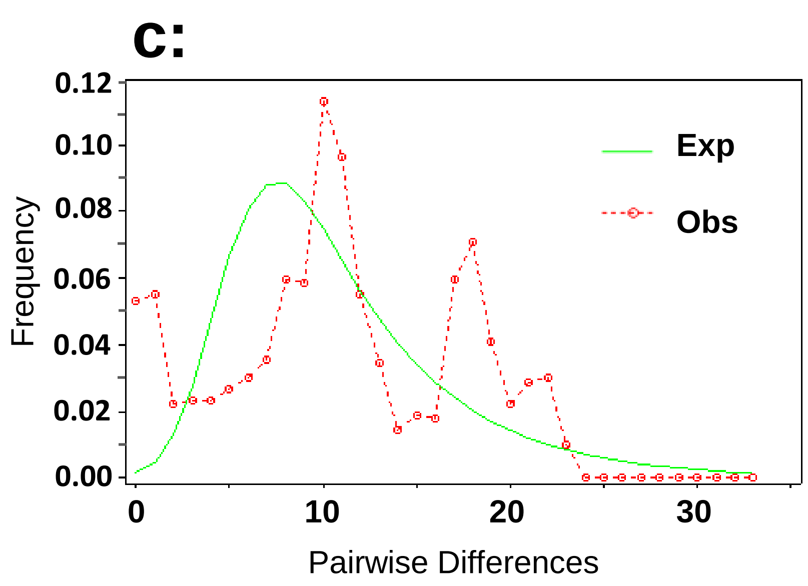

Additional file 2: Figure S2. Observed (Obs) and expected (Exp) mismatch distributions showing the frequencies of pairwise differences. The observed distributions (red dot lines) are compared for their goodness-of-fit to a Poisson distribution under a model of sudden expansion illustrated by the overlaid curve (green solid lines). **a**, All populations; **b**, All populations without Yunnan; and **c**, Two populations from Yunnan province.
